# *Spiculopteragia boehmi* is the dominant abomasal nematode species in reindeer (Nordland County, Norway) sharing pasture with wild and domesticated ruminants

**DOI:** 10.1186/s13028-026-00853-w

**Published:** 2026-02-10

**Authors:** Elle Káre Eira, Terje Domaas Josefsen, Tsegabirhan Kifleyohannes, Kjersti Selstad Utaaker

**Affiliations:** 1 University of Veterinary Medicine and Pharmacy in Košice, Košice, 041 81 Slovakia; 2 Faculty of Bioscience and Aquaculture, Nord University, Bodø, N-8026 Norway; 3 ParasitologyDepartment of Paraclinical SciencesFaculty of Veterinary Medicine, Norwegian University of Life Sciences, Ås, N-1432 Norway; 4 Norwegian Veterinary Institute, P.O. box 64, Ås, N-1431 Norway

**Keywords:** Abomasal nematodes, *Elaphostrongylus rangiferi*, Gastrointestinal parasites, *Giardia duodenalis*, *Rangifer tarandus*

## Abstract

**Background:**

Gastrointestinal parasites, especially those in the abomasum, are considered important production-limiting parasites of ruminants. Reindeer harbour many species of gastrointestinal parasites, and *Ostertagia gruehneri*, considered their dominant abomasal parasite, has been relatively extensively studied. This study aimed to assess the prevalence, species composition, and burdens of gastrointestinal parasites in a relatively southern flock of semi-domesticated reindeer in Duokta, Norway. In Duokta, a lower number of reindeer than domestic sheep share pastures, alongside an increasing moose population and a relatively new roe deer population. In the present study, visceral and faecal samples were collected during the winter slaughter of 47 semi-domesticated reindeer in 2020 in a local slaughterhouse in Duokta. The samples were analysed qualitatively and quantitatively.

**Results:**

From the 47 animals, 16 visceral and 42 faecal samples were collected. Subsamples of the abomasum were checked for numbers and species of nematodes. A McMaster method, Baermann technique, and a direct immunofluorescent antibody test was used to analyse the faecal samples. Four nematode species were detected from the abomasum samples (*Spiculopteragia boehmi*,* Ostertagia gruehneri*,* Teladorsagia circumcincta* and *Mazamastrongylus dagestanica).* All of the animals had low numbers of eggs, oocysts or cysts of at least one parasite species in their faeces, whereas higher numbers of larvae were found. Molecular analysis revealed *Giardia duodenalis* Assemblage A and B. Both have zoonotic potential and the latter is not previously reported from semi-domesticated reindeer.

**Conclusions:**

The abomasal nematode fauna was dominated by *Spiculopteragia boehmi*, a species of uncertain significance, though no apparent impact on the slaughter weights was observed. The faecal egg counts was not correlated with the abomasal nematode counts, underscoring the importance of considering season of sampling. The brainworm *Elaphostrongylus rangiferi* was found at a high, but not exceptional, prevalence in reindeer older than 1.5 years, consistent with previous studies from Norway. Zoonotic *Giardia* assemblages may indicate potential for anthropozoonotic transmission.

**Supplementary Information:**

The online version contains supplementary material available at 10.1186/s13028-026-00853-w.

## Background

Reindeer (*Rangifer tarandus*) are medium-sized ungulates found in the northern hemisphere of America, Asia, and Europe [1]. There are two varieties of reindeer management in mainland Norway: wild reindeer and semi-domesticated reindeer.

Semi-domesticated reindeer are an essential source of livelihood throughout northern Fennoscandia, are vital to the owner’s economy and are important food and raw material sources [2].

In Norway, this husbandry is mainly practised in the Sami areas, ranging from Finnmark in the north to Engerdal in Innlandet in the south.

There were 213,000 semi-domesticated reindeer throughout northern Norway in March 2021, with the majority living in the two northernmost counties, Troms and Finnmark. Reindeer husbandry is also practiced in Nordland, where the animal count was 14,400 in March 2021, as opposed to 147,000 in Finnmark [3]. Semi-domesticated reindeer in Nordland share pastures with an increasing population of moose (*Alces alces*) [4] and roe deer (*Capreolus capreolus*) [5]. There are also 73,500 domestic sheep in Nordland, compared to 9,600 in Finnmark, who graze freely on rangeland during the summer [6].

The natural pasture areas in Duokta are decreasing as a result of human activities and climate change [7]. Reindeer share these areas with both wild and domestic ruminants, who may transmit pathogens between them [8]. Gastrointestinal parasite transmission is particularly influenced by warm and humid conditions, which favour the survival and spread of parasite larvae. The likelihood of these parasites infecting a host increase when susceptible animals graze on shrinking pastures.

Transmission of parasites between sympatric hosts is well-studied and documented for co-grazers in many regions [9–11]. Though recent studies are lacking for Norwegian semi-domesticated reindeer, which could be due to their dominance in the more northern parts of Norway where fewer alternative hosts are present.

In Duokta, semi-domesticated reindeer are outnumbered by other grazing species which could influence parasite transmission dynamics.

Semi-domesticated reindeer are nomadic and are only gathered a few times a year, primarily during separation of different flocks and for slaughter. These gatherings usually occur during periods when the animals are not calving, nor during the rut, but when animals are seemingly not immunosuppressed or stressed. Thus, parasite shedding of transmission stages is presumably low, making faecal helminth egg counts from sampling on these occasions an unreliable indicator of actual parasite burdens. Additionally, many gastrointestinal nematodes produce strongyle-type eggs, which cannot be differentiated at the species level through a conventional faecal analysis. Assessing the abomasal parasite fauna in ruminants using traditional methods is labour-intensive and requires specialized skills to accurately identify nematode species. Newer technologies, such as metabarcoding, do provide a relative indication of parasite species presence, though does not accurately reflect the true parasite abundance, viability or life stage [12].

Studies on abomasal parasites in reindeer have primarily been conducted in the wild reindeer populations in southern Norway [13–15]. A master thesis from 2020 [13] also included a subset of samples from reindeer in Finnmark. Until recently, the only study on the nematode fauna of semi-domesticated reindeer in Norway dated back more than 40 years [16] and was conducted in Troms, a more northern county than Nordland. Since then, a recent master thesis [17] has provided new data on nematode communities in semi-domesticated reindeer, further contributing to the knowledge of geographic variation.

This study aims to assess the parasite fauna in winter-slaughtered semi-domesticated reindeer in Duokta, to investigate potential parasite spillover and transmission dynamics between sympatric species. Traditional parasitological methods were applied to examine abomasal parasites, faecal egg/oocyst counts (McMaster method), larval isolation (Baermann technique), and immunofluorescence assays (IFAT) for *Giardia* and *Cryptosporidium*. By establishing baseline data on parasite presence and diversity, this study contributes to understanding how shared grazing areas influence parasite transmission in semi-domesticated reindeer in a relatively southern reindeer husbandry district.

## Methods

### Study area

**Fig. 1:**
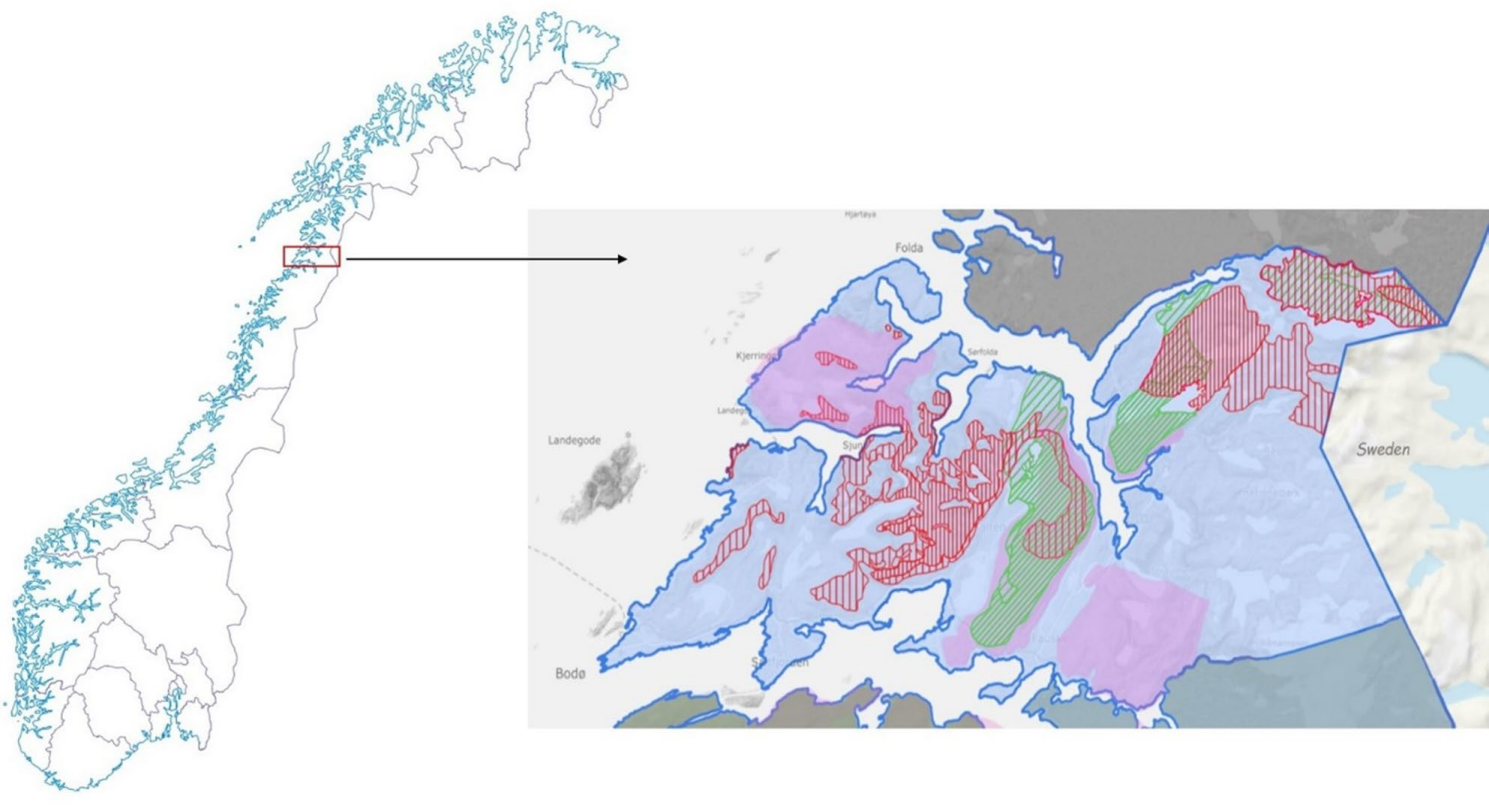
District 26 Duokta, outlined in blue. Legend: The red-striped areas are the summer pasture for reindeer, the narrow-striped areas are grazing areas in higher altitudes, and the wider-striped areas are grazing areas close in lower altitudes. The green striped areas are the autumn pasture for reindeer, where the narrow-striped areas are grazing areas in the early autumn season and the wider striped areas are grazing areas during the mating season. The pink areas are sheep pastures. The illustration is obtained with data from Kartverket 2025 [21] and Norsk institutt for bioøkonomi [22]

In Reindeer district 26, Duokta (Fig. 1), about 700 semi-domesticated reindeer [3] share pastures with an increasing moose and roe deer population [18, 19] and 3,000 domestic sheep [20]. Duokta is located in Bodø, Fauske and Sørfold municipality east of Skjerstadfjorden and on Kjerringøy in Nordland County.

### Collection of samples

The Duokta herd was gathered from the winter grazing area for treatment with ivermectin and sorting animals for slaughter. Animals sorted for slaughter were not treated with anthelmintics. Sampling was done on 9 December (early winter) 2020 at a local reindeer slaughterhouse. Sampling was performed coherently with the slaughter and samples were marked chronologically with a number corresponding to the slaughterhouse journal, so that the sex, age class and carcass weight of the sampled animals could be provided by the slaughterhouse (Additional file 1). In this study we chose to distribute the slaughterhouse classes into two age categories: calves and adults (> 1.5 years).

#### Abomasum

The abomasa were collected immediately after evisceration of the gastrointestinal tract from the first 16 animals slaughtered. Cable ties were used to ligate the junction between the omasum and the abomasum and the proximal part of the duodenum. The omasum and distal part of the ligated duodenum were cut using scissors, and the separated ligated viscera was put in marked plastic bags.

#### Faeces

Faecal samples were collected directly from the rectum of the viscera from slaughtered reindeer. Five of the 47 animals had no faeces in the rectum and were excluded from the faecal analyses. The remaining 42 faecal samples were put into numbered sample containers.

The samples were transported to Nord University in Bodø on the day of collection. The faecal samples were kept in the fridge at 4 °C, while the visceral samples were frozen at -12 °C before processing and analysis.

### Parasitological procedures

#### Abomasum

The abomasa were thawed at 4 °C, cut open, and its content was placed in a bucket. The abomasal wall was washed with tap water, and the wash water was poured carefully into the bucket. This procedure was repeated until the total volume in the bucket was 2 L. While stirring the suspension, subsamples were collected in four 50 mL tubes (4 × 2.5% aliquots). Marked aliquots were stored in the freezer at -12 °C for later processing, or in the refrigerator at 4 °C.

For enumerating abomasal nematodes, an aliquot was poured into a 500 mL beaker, filled with approximately 200 mL of tap water, and left to sediment for 30 min. The supernatant was removed before adding up to 300 mL tap water to clear the suspension, depending on the amount of debris in the subsample. The suspension was poured on a gridded dish, and each grid was screened using a stereomicroscope at magnification 10x-16x (Leica M50 with Leica cold light source CLS 100X). An entomological pin was used to move debris which may cover the nematodes. Nematodes were collected and stored in 70% ethanol. Collection stopped at the aliquot where the nematode count reached 100 during counting or after collecting nematodes from 5% of the abomasal content.

The sex of the collected nematodes was determined by identifying the males’ bursal organs, whereas the females were counted and discarded. Only the male nematodes were used for species identification. For each species, the number of female nematodes was assumed to be proportionally equal to that of the males.

Male nematodes were placed on a microscope slide, and one drop of water was added before a coverslip was placed over the nematode. Species identification was performed using a Leica DM 1000 microscope at 100x, 200x, and 400x magnification. Males were speciated according to size and length, and male bursal organs such as spicules and bursal morphology (Additional file 2) [23–27]. When the nematode species was challenging to identify, a drop of polyvinyl lactophenol (Waldeck GmbH) was used instead of water to clear the nematode for easier identification.

### Faeces

#### McMaster flotation method

Faecal samples were analysed within a week of collection. A modified McMaster method was used to estimate the quantity and type of helminth eggs and coccidian oocysts in the faecal samples.

Three grams of faeces were homogenized with 57 mL of tap water. The suspension was filtered through a sieve (mesh size 800–1000 μm), collected into two 15 mL test tubes and centrifuged at 1,500 relative centrifugal force (RCF) for 3 min. The supernatant was discarded after centrifugation, and the pellet from one test tube was resuspended in Curavet^®^ (WDT, Germany) flotation solution. Two and a half mL of the suspension was placed into a counting chamber (Whitlock Universal, Australia). The whole slide was examined at 40x and 100x microscope magnification to detect and quantify helminth eggs and *Eimeria* oocysts, with a detection limit of 10 eggs/oocysts per gram faeces (EPG/OPG).

#### Baermann method

The faecal samples were covered with gauze, forming a pouch that was attached to a stick and placed in a funnel. The funnel was filled with lukewarm water until it covered the sample and left for a minimum of 18 h. The water in the funnel’s neck was collected and transferred to 15 mL tubes and centrifuged for 5 min at 1,500 RCF. The supernatant was removed, leaving 1 mL of water and sediment in the tube. The fluid and sediment were mixed thoroughly, and a 100µL subsample of the suspension was examined with a microscope at 100x magnification. L1 larvae were identified and counted. The subsample count was used to estimate the number of larvae per gram faeces (LPG) with a theoretical detection limit of 1 LPG in 10 g samples (number of larvae detected in 100 µL x 10/the weight of the faeces in the faecal sample) [27].

If no larvae were observed in the 100 µL subsample, the centrifugation tube was left to sediment for about 30 min, and 100µL of precipitate was again examined. If this precipitate sample was also negative, the sample was stated as negative. If larvae were observed in the precipitate sample, the sample was stated as positive and LPG was set to 1.

L1 larvae with protostrongylid morphology (kinked tail with dorsal spine) were identified as *Elaphostrongylus* sp., as no other protostrongylid species are known to occur in reindeer in Europe [28]. The preferred amount of faeces for Baermann method was 10 g from each animal. However, faecal samples were often too small to achieve this, and samples down to 2 g were accepted. McMaster flotation was prioritized, resulting in no faecal material left for Baermann method in six reindeer. Samples were 10 g in eleven animals, 6–9 g in 15 animals and 2–5 g in seven animals. Three reindeer had to be excluded as they had loose faeces (diarrhoea) that were impossible to wrap in gauze. Thus, the total number of animals examined by Baermann method was 33.

Immunofluorescent antibody test (IFAT).

The immunofluorescence antibody test (IFAT) was used to investigate the presence of *Giardia duodenalis* cysts and *Cryptosporidium* oocysts on direct faecal smears. An inoculation loop placed 5–20 µL of homogenized and sieved faeces on a microscope slide. Airdried slides were fixated with a drop of methanol and labelled with 15–20 µL of FITC-labelled monoclonal antibodies (Mab) against *Giardia* and *Cryptosporidium* cyst walls (Aqua-Glo; Waterborne Inc., NO, USA). After incubation in a humid chamber for 45 min, excess Mab was removed with distilled water and a coverslip was added. Fluorescence microscopy was used to examine the stained smears under the following settings: FITC: emission 490 nm, excitation 525 nm, at x200 and x400 magnification. Positive samples were graded semi-quantitatively by counting the number of cysts per field view at x200 magnification.

### Molecular methods

#### DNA isolation

*Giardia*–positive samples were selected for molecular characterization by polymerase chain reactions (PCR). According to the manufacturer’s instructions, DNA was extracted from the cysts by PowerSoil^®^ DNA isolation kit (Qiagen, USA), except that FastPrep24^®^ 5G (MP Biomedical, USA) was used for bead-beating, which was done twice at 4 m/s for 60 s. The isolation and PCR was performed at the Parasitology Unit at the Norwegian University of Life Sciences, Ås.

### PCR, electrophoresis, purification of PCR product, and sequencing

Two genes were targeted for genotyping investigations of the isolated *Giardia* DNA; the β-giardin (*bg*) gene and the glutamate dehydrogenase (*gdh*) gene (Additional file 3) [29, 30].

#### Polymerase chain reaction and sequencing

The primary PCR included 8.3 µL PCR water, 12.5 µL of 2× DreamTaq Green PCR Master Mix (Thermo Scientific), 1 µL each of forward and reverse primers (0.1 mM), 0.2 µL BSA (20 mg/L) and 2 µL template DNA. Positive (P101, *G.duodenalis* cysts, human isolate H-3, Assemblage B, Waterborne Inc, LA, USA) and negative controls (lab-grade purified water) were included. SYBR™ Safe DNA Gel Stain (Life Technologies, CA, USA) was used to visualize PCR products after electrophoresis on 2% agarose gel. Upon positive results, the DNA amplicons were purified using ExoSAP-IT™ PCR Product Cleanup Reagent (Affymetrix USB, OH, USA) and sequenced in both directions. Purified products were sent to Eurofins Genomics in Germany for sequencing. Sequences were examined, assembled, and manually corrected by analysing the chromatograms using Geneious Prime^®^ 2022.0.2 software (New Zealand). Sequence comparisons were conducted using the National Center for Biotechnology Information Basic Local Alignment Tool (NCBI BLAST, MD, USA). Sequences were submitted to GenBank^®^ (see results section).

### Statistics

A database of results was created in Microsoft Excel, where also calculations of descriptive statistics and Spearman’s correlation test for association between female nematode counts and strongyle-type egg counts were performed. The online statistical tool VassarStats were used for calculating confidence intervals of parasite eggs, oocysts, cysts and larvae, and Mann-Whitney U-test was used for comparing total nematode counts as well as the different nematode species count between nematode species and age groups [31, 32].

## Results

### Gastrointestinal parasites

#### Adult nematodes in the abomasum

Abomasal nematodes were found in all calves (*n* = 7) and adults (*n* = 9, Table 1). *Spiculopteragia boehmi* and its minor morph, *Spiculopteragia mathevossiani*, were found in 94% of the samples, *Ostertagia gruehneri* was detected in 81%, *Teladorsagia circumcincta* was detected in 19%, and *Mazamastrongylus dagestanica* was found in one sample.

**Table 1:** Occurrence and intensity of adult nematodes in the abomasum

	*Adults*				*Calves*			
	*n*-positive/*n*-total	Median	Mean	Min-max	*n*-positive/*n*-total	Median	Mean	Min-max
**Species**								
*S. boehmi*	8/9	1931	2070	417–4270	7/7	463	493	60–1063
*S. mathevossiani*	5/9	70	157	44–508	4/7	95	92	48 – 130
*O.gruehneri*	8/9	960	956	102–1927	5/7	62	105	48–260
*T. circumcincta*	1/9	102	102	-	2/7	82	82	60–103
*M. dagestanica*	1/9	282	282	-	0/7	-	-	-
**Total nematode counts**	**9/9**	**3015**	**3041**	**487–4880**	**7/7**	**600**	**640**	**180–1159**

Varying lengths of duodenum were also incidentally included. These sections were cut longitudinally and washed in a strainer with mesh size 800–1,000 μm. Visible nematodes were collected using tweezers and stored in 70% ethanol, followed by speciation according to male bursal organs [25, 33]. *Nematodirus tarandi* were found in two samples, and *Bunostomum trigonocephalum* in one sample (Additional file 2).

Legend: A boxplot figure showing the intensity of the different nematode species in calves and adults.

Abomasal nematode counts, species and animal age.

**Fig. 2:**
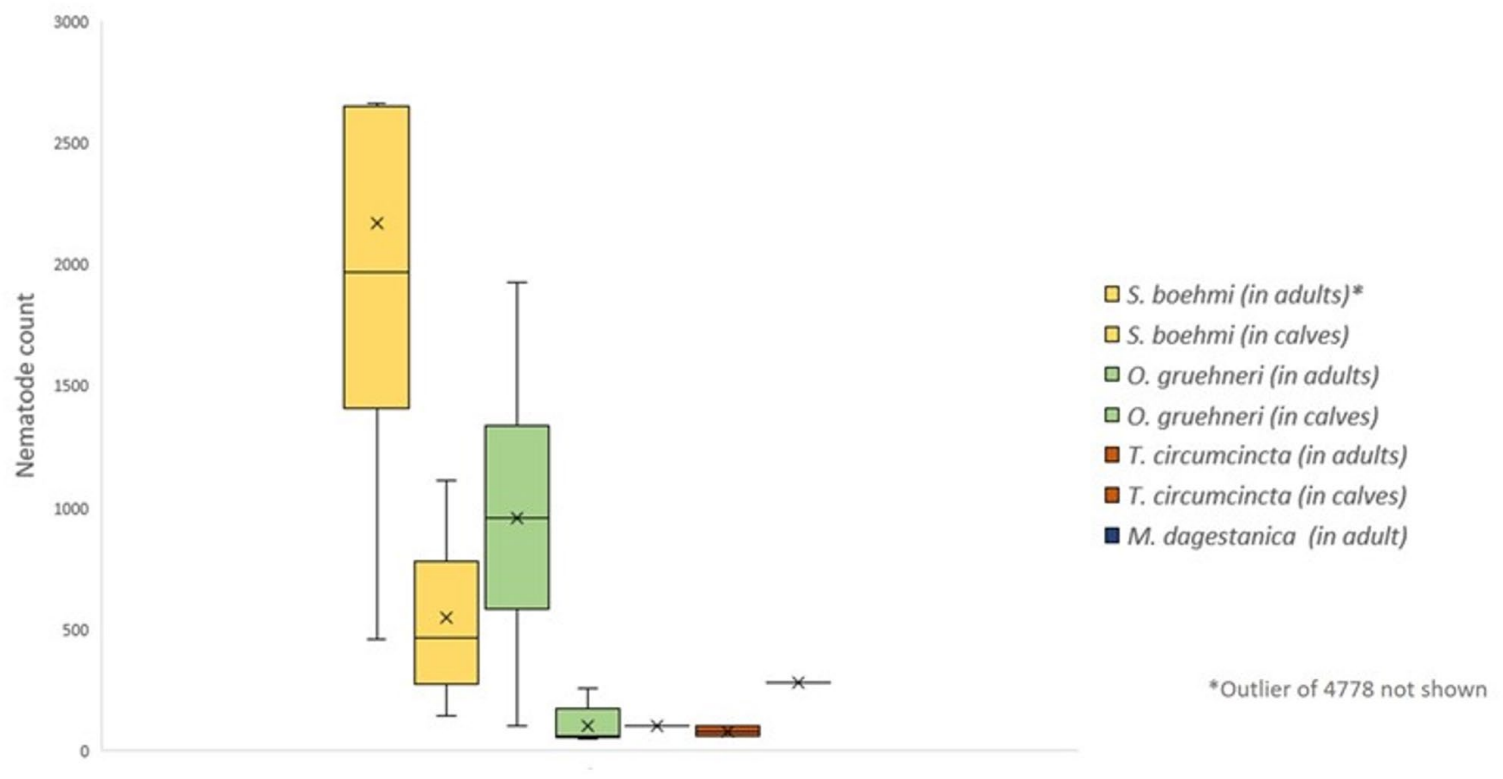
Count of each nematode species in adults and calves. Legend: A boxplot figure showing the intensity of the different nematode species in calves and adults

Figure 2 illustrates the amount of each nematode species in adults and calves.

The total nematode counts for adults and calves (Table 1) differed significantly. The different species numbers in the age groups were also compared (Table 1; Fig. 2) where *O. gruehneri* and *S.boehmi* were significant, while *T. circumcincta* and *M. dagestanica* were not significant.

The abundances of *S.boehmi* and *O.gruehneri* within all age groups was statistically significant.

There was no statistically significant correlation between the number of eggs and the estimated number of abomasal nematode females (Spearman’s rho = -0,21, *P* = 0.49).

### Findings from faecal analysis

All 42 (100%) of the animals had eggs, oocysts, cysts or larvae of at least one parasite species in their faeces. 27 (64%) of the 42 reindeer had a mixed infection with 2 − 6 different gastrointestinal parasite species, and seven types (species/genera/subfamily/order) of parasites were detected from the faecal samples (further details are shown in Table 2).

**Table 2:** Prevalence of parasite eggs, oocysts, cysts and larvae from McMaster, IFAT, and Baermann analysis

*n* total = 42 *n* calves = 12 *n* adults = 30	*n* positive (% positive)	95% CI %	Median EPG/OPG/LPG	Range EPG/OPG/LPG
McMaster (n = 42)				
strongyle-type eggs	37 (88.1)	73.6–95.5	40	10–250
strongyle-type eggs (calves)	9 (75)	50.9–97	50	10–80
strongyle-type eggs (adults)	28 (93.3)	72.3–97.4	20	10–230
Nematodirinae spp.	7 (16.7)	7.5–32	20	10–40
Nematodirinae spp. (calves)	4 (33.3)	11.3–64.6	15	10–20
Nematodirinae spp. (adults)	3 (10)	2.6–27.7	19	10–40
*Aonchotheca* sp.	19 (45.2%)	30.2–61.2	20	10–250
*Aonchotheca* sp. (calves)	3 (25)	6.7–57.2	20	10–250
*Aonchotheca* sp. (adults)	16 (53.3)	34.6–71.2	20	10–230
*Trichuris* sp.	5 (11.9)	4.5–26.4	10	10
*Trichuris* sp. (calves)	1 (8.3)	0.4–40.2	10	10
*Trichuris* sp. (adults)	4 (13.3)	4.4–31.6	10	10
*Eimeria* spp.	6 (14.3)	6–29.2	200	200 − 1,200
*Eimeria* spp. (calves)	1 (8.3)	0.4–40.2	200	200
*Eimeria* spp. (adults)	5 (16.67)	6.3–35.5	200	18–1,200
IFAT (*n* = 42*)*				
*G. duodenalis*	5 (11.9)	4.5–26.4		G+
*G. duodenalis* (calves)	3 (25)	6.7–57.2		G+
*G. duodenalis* (adults)	2 (6.7)	1.2–23.5		G+
*Cryptosporidium sp.*	0	0–0.1		
Baermann (*n* = 33) n calves = 10 n adults = 23				
*Elaphostrongylus* sp.	19 (57.6)	39.4–74	67	1–750
*Elaphostrongylus* sp. (calves)	3 (30)	8.1–64.6	67	44–413
*Elaphostrongylus* sp. (adults)	16 (70)	47–86	68	1-750

* EPG = Eggs per gram, OPG = oocysts per gram, LPG = larvae per gram faeces

### PCR analysis of giardia cysts

All positive samples had a low number of cysts, with only 1–9 cysts per field view at 200x magnification. DNA was isolated, and PCR was performed for all 5 samples, and positive results were obtained from 4. The two genetic loci gave the following positive results: 4/5 from the *gdh* gene and 2/5 from the *bg* gene. Of the sequences obtained, three belonged to Assemblage A, sub-assemblage AI, and one belonged to Assemblage B. The sequences have been deposited in GenBank under the Accession Numbers ON098387 - ON098390 for the *gdh* sequences and ON148008 - ON148009 for the *bg* sequences.

### *Cryptosporidium* sp.

No *Cryptosporidium* sp. oocysts were found in the samples.

## Discussion

Interestingly, this study found that the dominating abomasal nematode was *Spiculopteragia boehmi*. Although *S. boehmi* is known by multiple names; *Spiculopteragia boehmi* (Gebauer, 1932) syn. *Spiculopteragia spiculoptera* (Gushanskaya, 1931); *S. kutkascheni* (Assadov, 1952); *S. pigulevski* (Ruchliadev, 1961) and its minor morph, *Spiculopteragia mathevossiani* (Ruchliadev, 1948) syn. *Rinadia schulzi* (Grigoryan, 1951); *S. caucasica* (Assadov, 1955); *S. pavloskyi* (Kadenatsii & Andreeva, 1957); *S. quadrifurcata* (Andrews, 1964) [34–36], there are few reports on its life strategies and pathogenicity.

*S. boehmi* is primarily described as a parasite of red deer (*Cervus elaphus*), though it exhibits a broad host range among wild cervids [37]. Inoculation studies using *S. boehmi* larvae sourced from moose and reindeer faeces have resulted in patent infections in domestic cattle calves and sheep lambs [38]. Furthermore, the parasite has successfully transitioned from roe deer and red deer to European bison (*Bison bonasus*) [39], demonstrating its adaptability to various hosts. In Norway, *S. boehmi* has been found in red deer [40], in small proportions in moose [41] and in wild and semi-domesticated reindeer [13, 15, 16, 17], however, it was not the dominating species in the abomasum.

Unlike *O. gruehneri*, which may undergo hypobiosis when infecting reindeer [42], *S. boehmi* does not necessarily require hypobiosis as a part of its life cycle. A study of nematodes in roe deer shot or found dead throughout a year from two Polish regions [43] revealed that *S. boehmi* was dominant in one population and less prevalent in another. This distribution pattern was thought to be influenced by the presence of other sympatric grazing species [43].

Seasonal variations in *S. boehmi* abundance were also observed in the same study [43], with the lowest levels recorded in January and a marked increase beginning in May, peaking in August. The subsequent decline in October suggests that a portion of the population may enter hypobiosis, leading to reduced numbers in the abomasum. The study did not investigate potential clinical effects of the parasite.

In North America, *S. boehmi* is well-established in game-ranched reindeer populations in Canada, where it has been found to significantly impact body condition [44]. Its potential expansion into caribou populations in the temperate Nearctic region has been predicted, though establishment at Arctic latitudes remained uncertain [45]. The dominance of *S. boehmi* in reindeer at Duokta (67° latitude) suggests initial cross-species transmission due to shared grazing areas in the region [46] and confirms its viability at high latitudes in the Palearctic, as well as its affinity for reindeer as a host.

Previous studies on abomasal fauna in semi-domesticated reindeer found *Ostertagia gruehneri* to be the most prevalent nematode, often with 100% prevalence and accounting for the majority of the average intensity [16, 47–49]. In Duokta, *Ostertagia gruehneri* was not the most abundant abomasal nematode, which may be due to the relatively low reindeer density in the study area compared to more northern counties in Norway, and the apparent increasing numbers of sympatric ruminants [4, 5, 18, 19].

*Mazamastrongylus dagestanica* and *Teladorsagia circumcincta* were detected in low numbers. *M. dagestanica* is commonly occurring in moose [48], whereas *T. circumcincta* is common in sheep [26]. Given the large moose population in Duokta [19] and the shared grazing areas with sheep, cross-species parasite transmission seemingly occurs at low levels for these nematodes.

Abomasal nematodes may impact reindeer health by reducing appetite, weight gain, and body condition even at low intensities [50–52]. Additionally, high parasite loads correlate with decreased fecundity and fertility in wild Svalbard reindeer [53, 54]. In this study, abomasal nematodes were detected across all age groups, with seemingly low to moderate burdens [55].As the samples were collected during the winter, numbers of nematodes are probably underestimated due to hypobiosis of nematode larvae.

The slaughter weights were, in all age groups where data was available (Calves, bulls 1–2 years and does > 2 years), higher than the national average [3]. Thus, it is not likely that intestinal parasites have a significant impact on the body condition of reindeer in Duokta.

Over the past two decades, moose and roe deer hunting in Nordland have increased [5, 19] reflecting growing populations of these species. The rise has been most noticeable in the harvest of older animals and males, which may reflect higher hunting pressure in response to population growth. This may explain some of the nematode species found in this study, as roe deer and moose are suitable hosts for *S. boehmi*, though its principal hosts are red deer. *S. boehmi* in roe deer has been associated with sympatry with red deer [56]. As red deer are not found in Doukta [57], the origin of this parasite in semi-domesticated reindeer is elusive.

*Bunostomum trigonocephalum* was found in the duodenum of one animal – a nematode mainly found in sheep [26]. *B. trigonocephalum* has only been discovered in low occurrences in reindeer in two previous studies from Finland and Sweden [58, 59]. The few findings of this nematode in reindeer may indicate that it is not a well-established parasite in reindeer and that these animals may be accidental hosts.

Parasite eggs, oocysts and cysts were found with a high occurrence, but with low numbers in all calves and adults.

The samples in this study were collected during winter when many nematodes undergo hypobiosis, and adult nematodes produce few eggs. Seasonal inhibition to avoid unfavourable environmental conditions is a known feature of abomasal nematodes in general, and arrested larval development of *O. gruehneri* in reindeer is consistent during the winter [47].

Our findings, though with limited samples, underscore the limitations of faecal egg counts from reindeer during winter, as the correlation between the number of strongyle-type eggs in the faeces and adult female nematodes in the abomasum was weak and not significant.

The overall prevalence and median of Nematodirinae eggs was lower than other reports [60, 61].The egg output of Nematodirinae have been found to be at its highest during the winter months after consecutive sampling for a period of two years [62]. The numbers found in this study should be interpreted with caution as they are based on a single sampling event.

*Aonchotheca* (previously *Capillaria*) eggs had a relatively high occurrence in the present study, which is in concordance with other findings [62].

There is limited data on *Trichuris* in reindeer, and its seasonal fluctuations are poorly understood. Low prevalences of *Trichuris* in reindeer have been reported [60], and the prevalence of this parasite in cervids in Europe varies greatly [63]. *Trichuris* eggs are resistant in the environment and eggs may accumulate if animals are kept on enclosed pastures.

*Eimeria* spp. infection are mainly seen in young animals [60, 64]. The positive samples in the present study belonged to adults, mostly those above 2.5 years. These samples contained a low number of oocysts, which is not an unusual observation in adult animals, who are generally immune to disease caused by *Eimeria* spp. at 1 year of age. The impact of *Eimeria* infection on reindeer is unknown, though clinical infections are not considered common under natural conditions [65]. A survey from 2019 [60] found numbers of *Eimeria* oocysts and *Trichuris* eggs in a few calves which could be of clinical importance and identified higher reindeer density as a risk factor for *Eimeria* infections. *Trichuris* egg and *Eimeria* oocyst numbers were noted as useful indicators for monitoring reindeer health when implementing husbandry changes [60].

The reindeer brainworm *Elaphostrongylus rangiferi* is common in all parts of the reindeer herding area in Norway [66]. The prevalence in the present study (70% in reindeer > 1.5 years) is at a comparatively high level [66], but not exceptionally high. Here, also samples as small as 2 g were included in the analysis, which may have led to false negatives for light infections. Prevalences ranging from 75 to 81% is reported in adult reindeer in Trøndelag County [67], and a prevalence of 100% has been reported in some years in Finnmark County [74]. Maximum LPG in the present study is 750, while 878-1,324 LPG is reported as maximum in different age classes from other flocks in Norway [67].

The lower prevalence in calves (30%) is explained by the life cycle of the parasite. The majority of infections occur in late summer or autumn [66], and the prepatent period is 4-4.5 months [68]. Thus, not all infected calves have started to shed larvae in December. Reindeer show seasonal variation in both prevalence and faecal density of *E. rangiferi* larvae [67, 70], both being at its lowest in summer (July-August), and generally high in winter (November-April). A distinct sex difference exists: Male reindeer peak in larvae output in December, after the rut, while female reindeer peak in February-April, in late pregnancy [69]. In the present study, the reindeer > 1.5 years sampled were 8 males and 15 females. Though the females are probably not in peak excretion of larvae, the prevalence can be considered fairly representative of the *E. rangiferi* infection level in the herd.

*Giardia duodenalis* assemblage AI appears to be the prevailing *Giardia* genotype in both wild and semi-domesticated reindeer [61, 71, 72]. The zoonotic (sub)Assemblages AI and B were detected in this study. AI is primarily found in animals, AII is commonly found in humans, and AIII only occurs in wild ungulates, while Assemblage B is predominantly found in humans [73]. The cyst counts in all positive samples were low, which may indicate asymptomatic infections or carriage. Assemblage B has previously been found in one Norwegian wild reindeer and in one reindeer sample of unknown origin [72].

*Cryptosporidium* sp. were not detected in the samples, which is not surprising as this parasite is mainly associated with animals younger than three weeks [74].

In the current study, all animals examined carried parasites or shed parasite eggs, larvae and/or oocysts and cysts at seemingly low to moderate levels, though the season of sampling was not optimal for assessing parasite burdens. All animals passed the ante- and post-mortem examination at the slaughterhouse and showed no signs of clinical illness. Although these animals carried nematodes which are not commonly associated with reindeer, they seemingly have had a low impact on their weight gains and overall health.

## Conclusion

This study revealed a diverse parasite fauna in semi-domesticated reindeer in Duokta. Parasite burdens were generally lower than those reported from Troms and Finnmark, reflecting lower reindeer density and effective herd management practices. Several parasite species of uncertain clinical significance for reindeer were identified. *Spiculopteragia boehmi* was identified as the dominant abomasal nematode, replacing *Ostertagia gruehneri*. This suggests possible transmission from other cervids, warranting further study of pasture overlap and host interactions, as well as molecular investigations of *S. boehmi* from different hosts for species assessment. *Elaphostrongylus rangiferi* was highly prevalent, emphasizing its significance in reindeer health and productivity. The identification of a zoonotic *Giardia* genotype in reindeer raises concerns about potential anthroponotic transmission. These findings underscore the complexity of parasite-host dynamics and the necessity for continued research into cross-species transmission, parasite ecology, and health implications for reindeer under changing environmental conditions.

## Supplementary Information

Below is the link to the electronic supplementary material.

Supplementary Material 1

Supplementary Material 2

Supplementary Material 3

## Data Availability

The dataset used during the current study are available from the corresponding author on reasonable request.
