# Supplementary material for: Spiculopteragia boehmi is the dominant abomasal nematode species in reindeer (Nordland County, Norway) sharing pasture with wild and domesticated ruminants
Source: Acta Vet Scand. 2026 Feb 10;68:10. doi: 10.1186/s13028-026-00853-w (PMC12896319; doi:10.1186/s13028-026-00853-w)
Supplement: Supplementary file 1 — Supplementary Material 1 [file 13028_2026_853_MOESM1_ESM.docx]

Additional file. 1. Age class, sex and carcass weight of reindeer sampled in this study, from the slaughterhouse journal.

| **Age*** | **No of animals** | | **Faecal samples** | **Visceral samples** | **Mean carcass weight (kg) [range]** |
| --- | --- | --- | --- | --- | --- |
| Calf (6-8 months) | | 13 | 12 | 7 | 21.0 [16.1 – 24.9] |
| Bull (1.5 -2 years) | | 8 | 8 | 4 | 32.2 [24.1 – 39.6] |
| Bull (>3,5 years) | | 2 | 2 |  | 47.1 [43.2 – 51.0] |
| Castrated Bull | | 3 | 3 | 1 | 61.9 [52.3 – 66.8] |
| Doe (1.5-2 years) | | 5 | 5 |  | 33.2 [30.7– 40.1] |
| Doe (>2.5 years) | | 12 | 12 | 4 | 41.5 [29.1– 67.9] |
| **Age category** | |  |  |  |  |
| Calf | | 13 | 12 | 7 | 21.0 [16.1 – 24.9] |
| Adult (>1.5 years) | | 30 | 30 | 9 | 39.5 [22.1 –67.9] |
| **Total samples** | **43** | | **42** | **16** |  |

*No bulls between 2,5 – 3 years were slaughtered on this day.
