# Supplementary material for: Spiculopteragia boehmi is the dominant abomasal nematode species in reindeer (Nordland County, Norway) sharing pasture with wild and domesticated ruminants
Source: Acta Vet Scand. 2026 Feb 10;68:10. doi: 10.1186/s13028-026-00853-w (PMC12896319; doi:10.1186/s13028-026-00853-w)
Supplement: Supplementary file 2 — Supplementary Material 2 [file 13028_2026_853_MOESM2_ESM.pdf]

*Spiculopteragia boehmi*

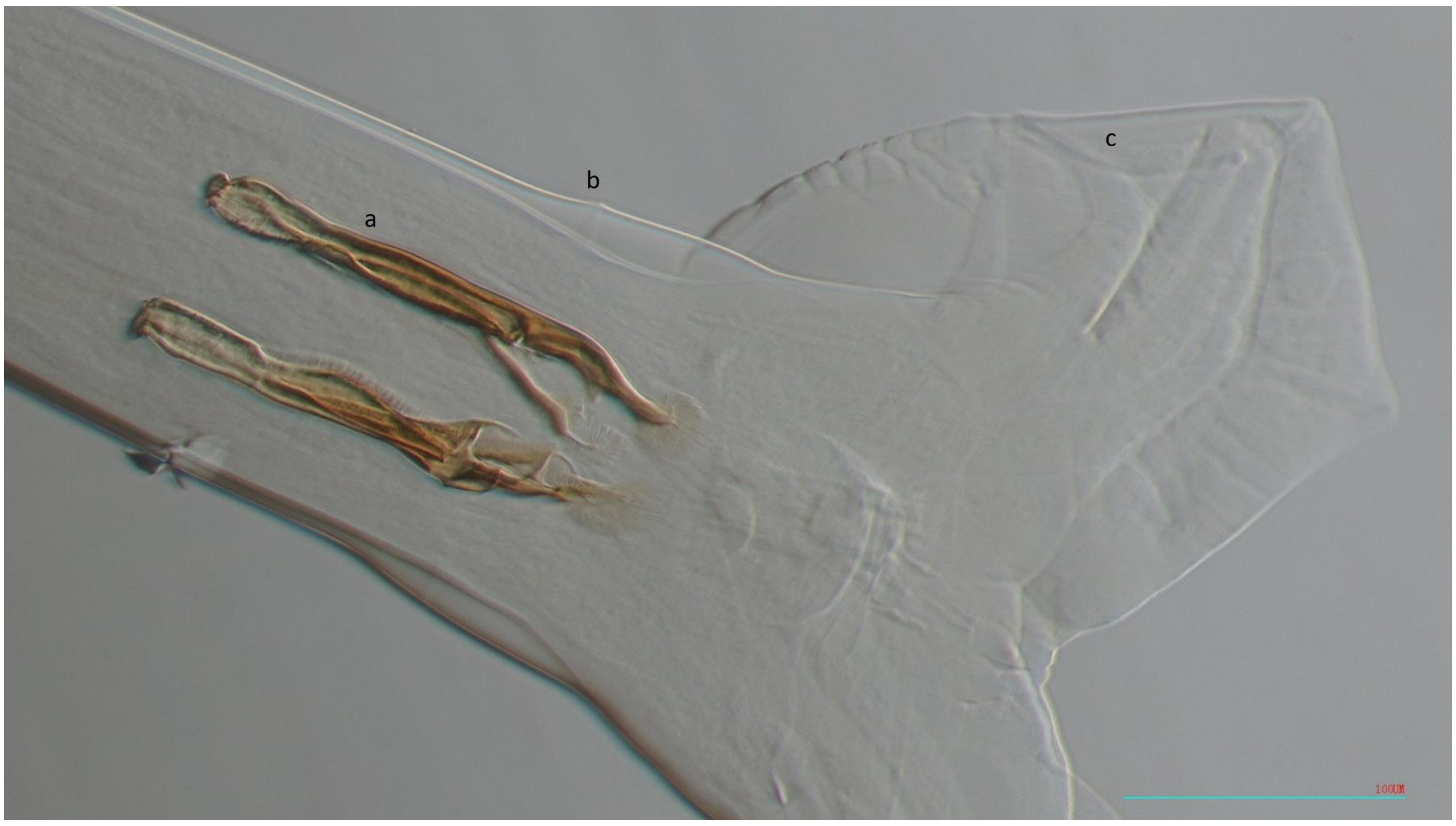


a) Asymmetrical spicules ~185µm long. left spicule divides into three branches, the right into two. Spicules ending in a fan-shaped expansion.
b) Prebursal papillae
c) Short thick anterolateral ray with blunt edge does not reach bursal edge. 2-2-1 pattern of bursal rays.

Gubernaculum absent.

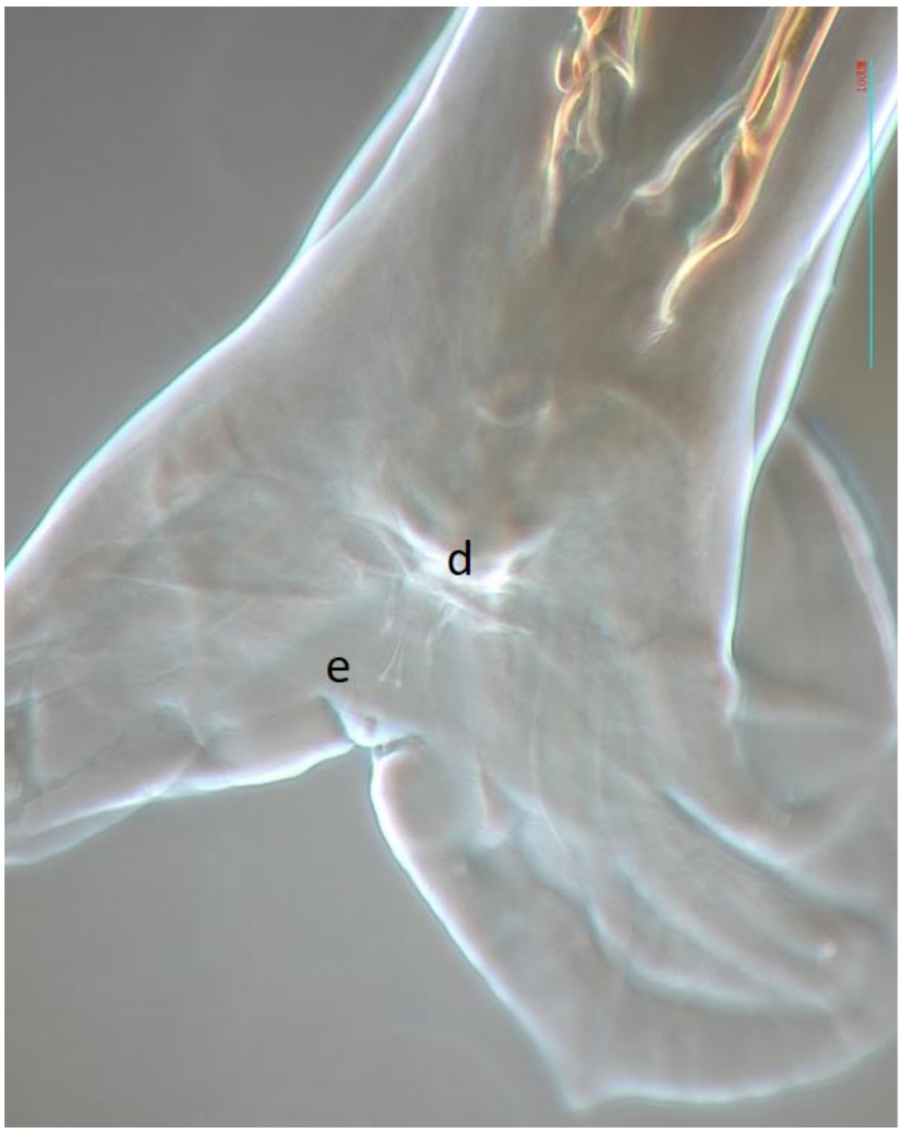


d) Telamon at ventral part of genital cone
e) Accessory bursal membrane with rays parallel and close together along most of their length. Small and separate dorsal lobe together with dorsal ray.

*Spiculopteragia mathevossiani* (minor morph *S.boehmi*)

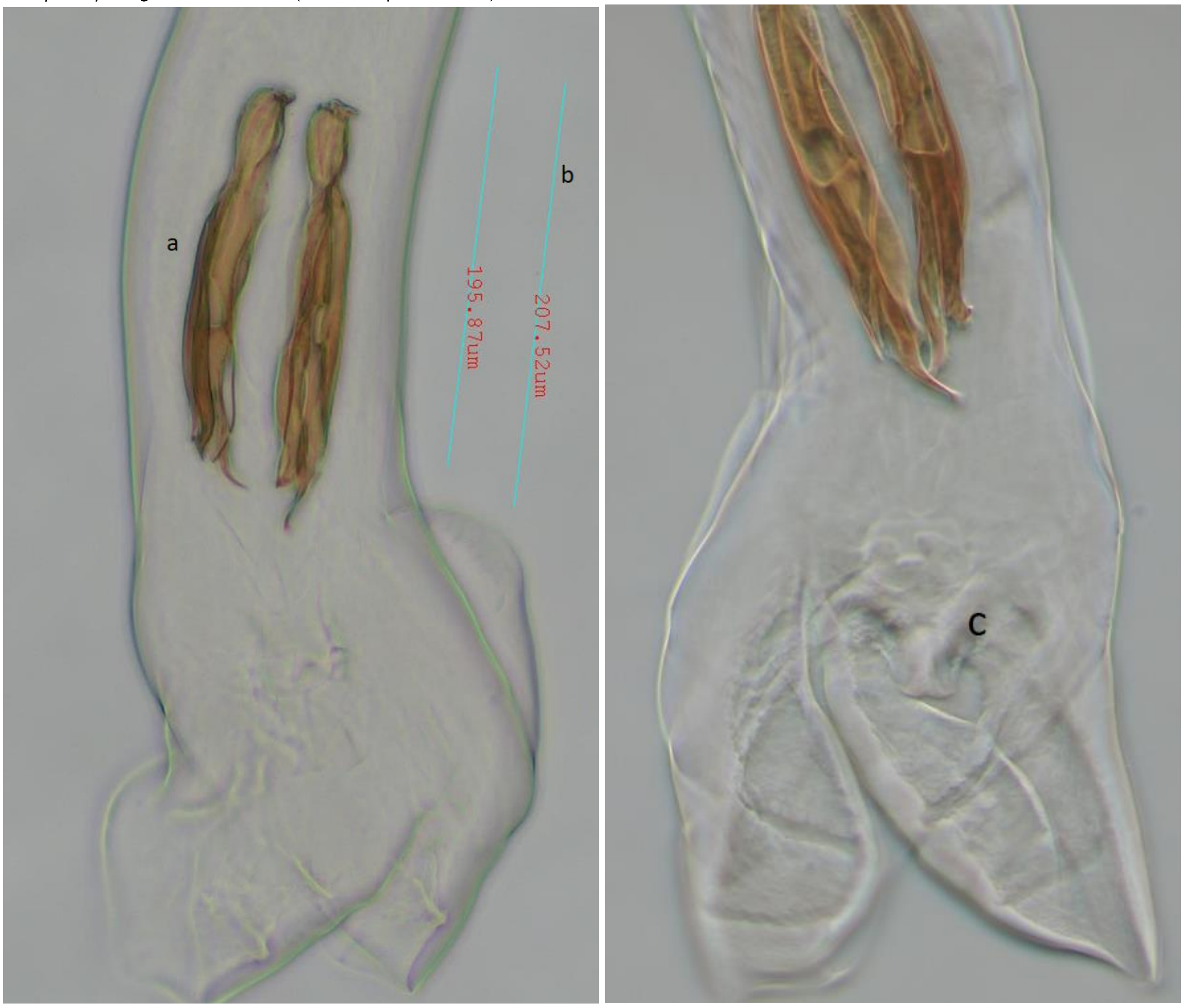


a) Spicules trifucated in the distal part
b) Length of spicules
c) Genital cone has a typical conical structure with a terminal bifurcation.

Prebursal papillae not visible, bursal rays not clear.

Gubernaculum absent

*Ostertagia* sp consistent with *O. gruehneri* based on the bursal structures

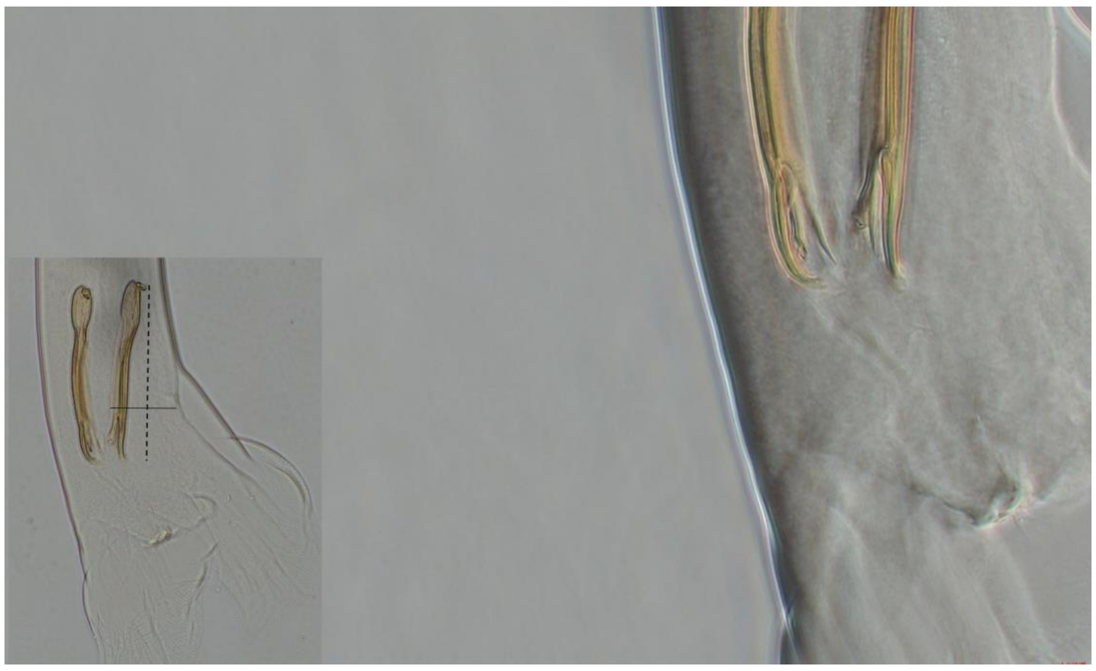

Spicules trifurcating in their distal third into an outer and two inner processes. Tip of spicule ends in sharp point.

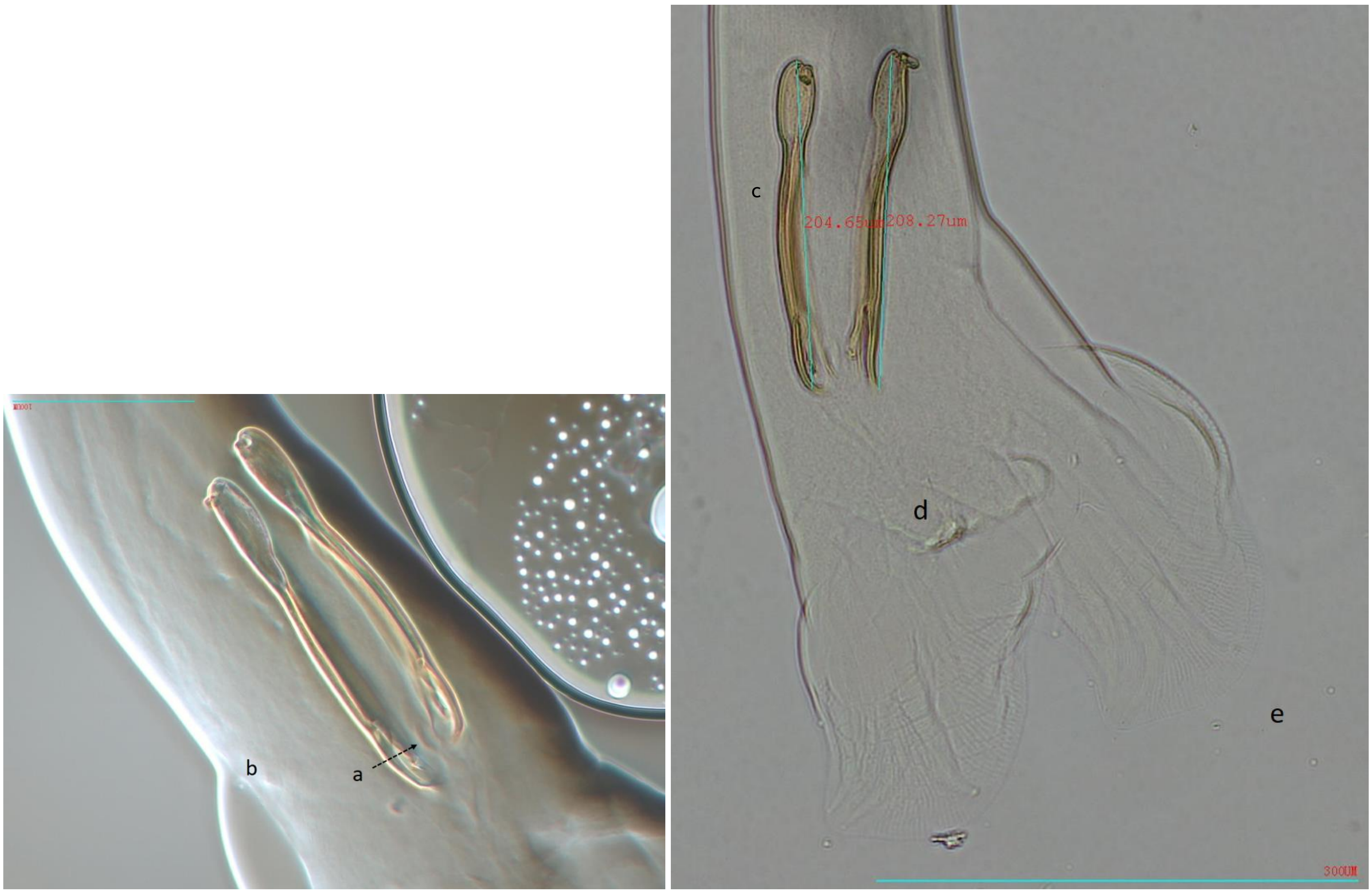


a) Gubernaculum
b) Prebursal papilla
c) Length of spicules
d) Genital cone
e) 2-1-2 arrangement of the bursal rays

# *Mazamastrongylus dagestanica*

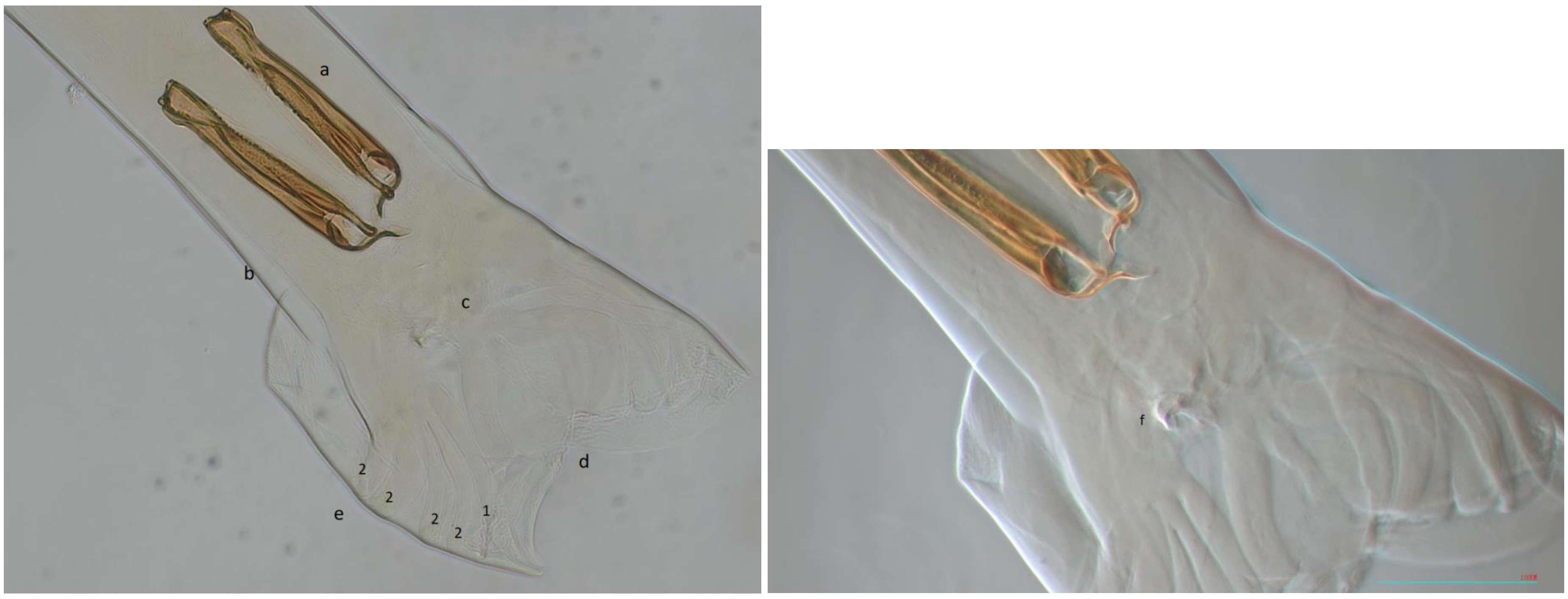


a) Typical symmetrical spicules with spoon-shaped dorsal processes and nods in the end.
b) Prebursal papillae
c) Genital cone
d) Bursa has two large lobes divided by a deep incicsion
e) 2-2-1 arrangement of the bursal rays
f) Telamon

Gubernaculum absent.

*Teladorsagia circumcincta*

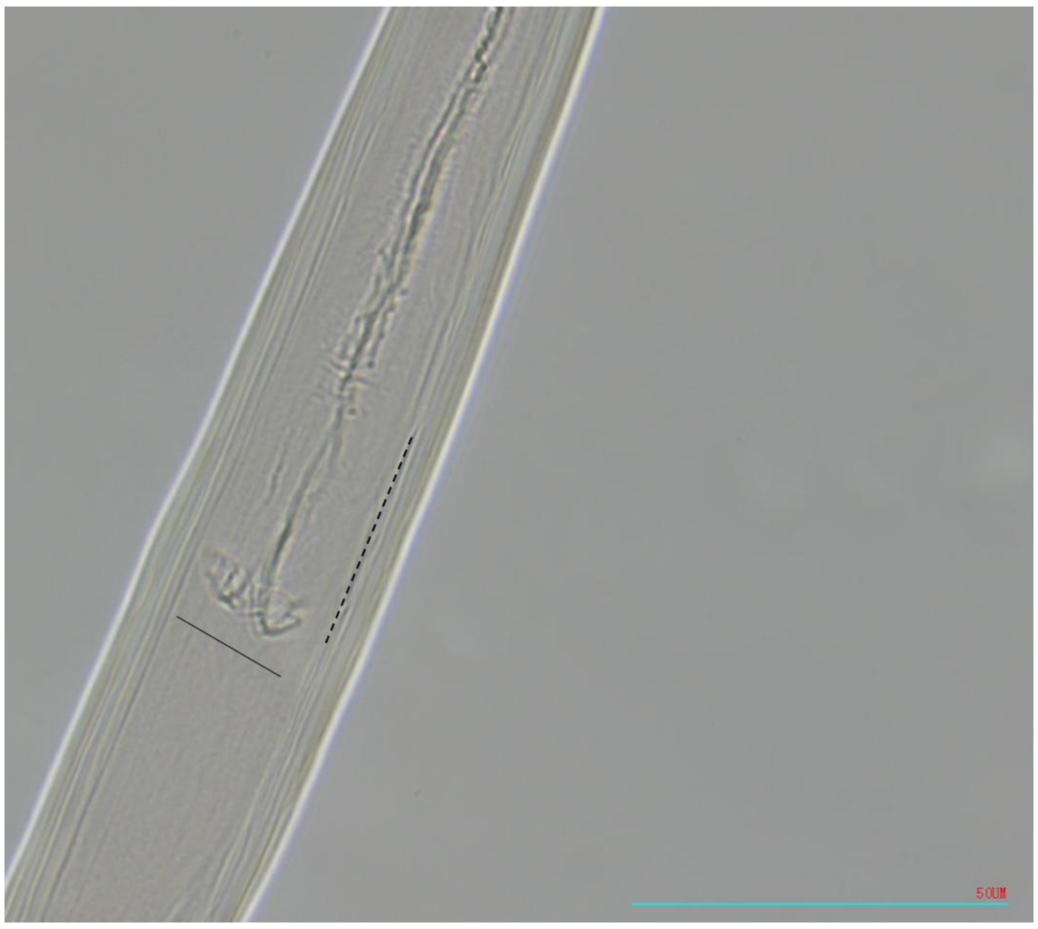


Esophageal valve shorter than twice its diameter

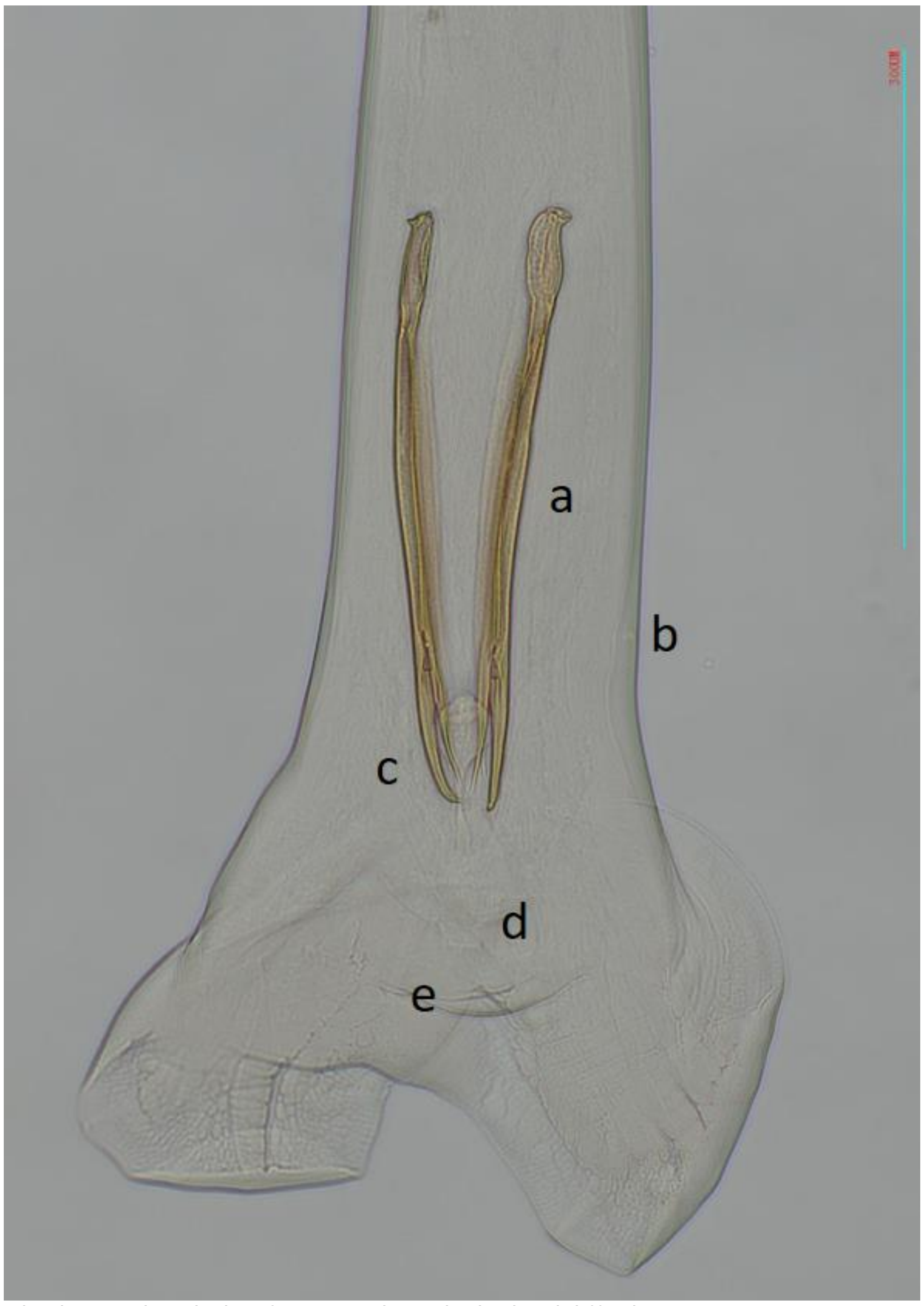


a) Slender spicules which trifurcate at the end. Third end difficult to see
b) Prebursal papillae
c) Gubernaculum is ~90μM long and has the shape of a tennis racket
d) Genital cone

e) Not very visble; dorsal ray bifurcates after 2/3 of its length

*Nematodirus tarandi*

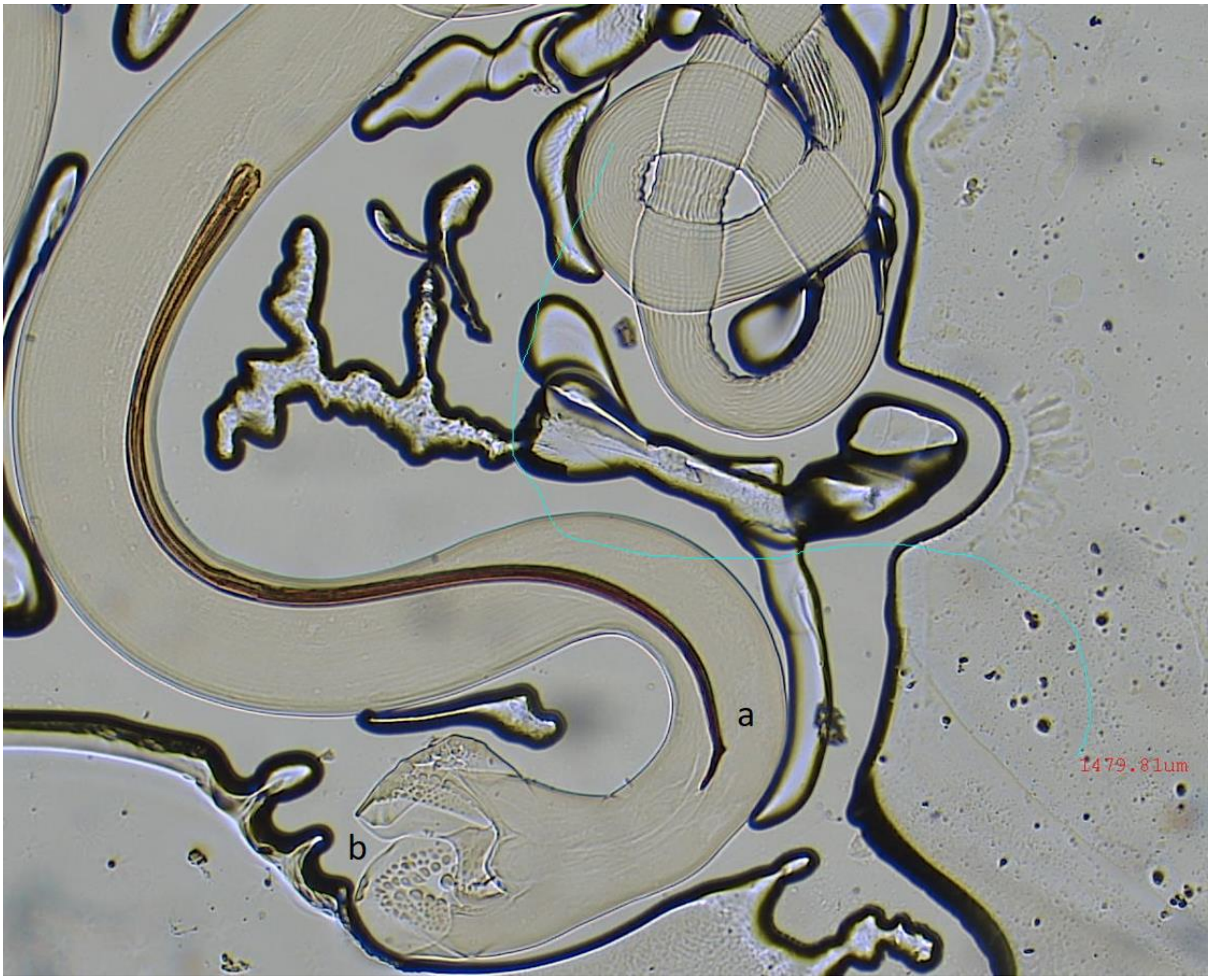


a) Spicules 1.3 - 1.6 mm long, terminating in a footlike process
b) Numerous bursal bosses

*Bunostomum trigonocephalum*

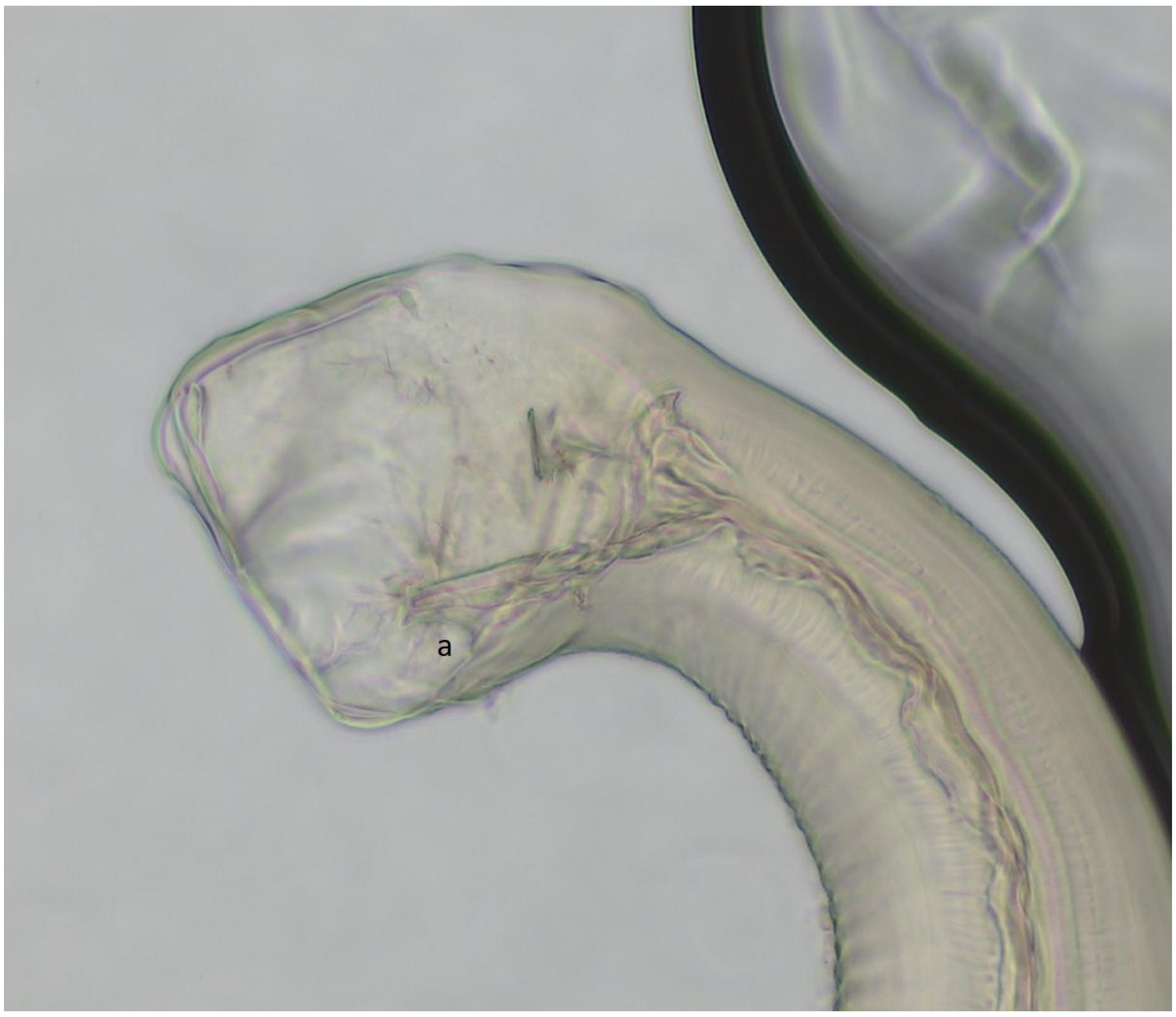


a) Buccal capsule with subventral lancets extending from bottom

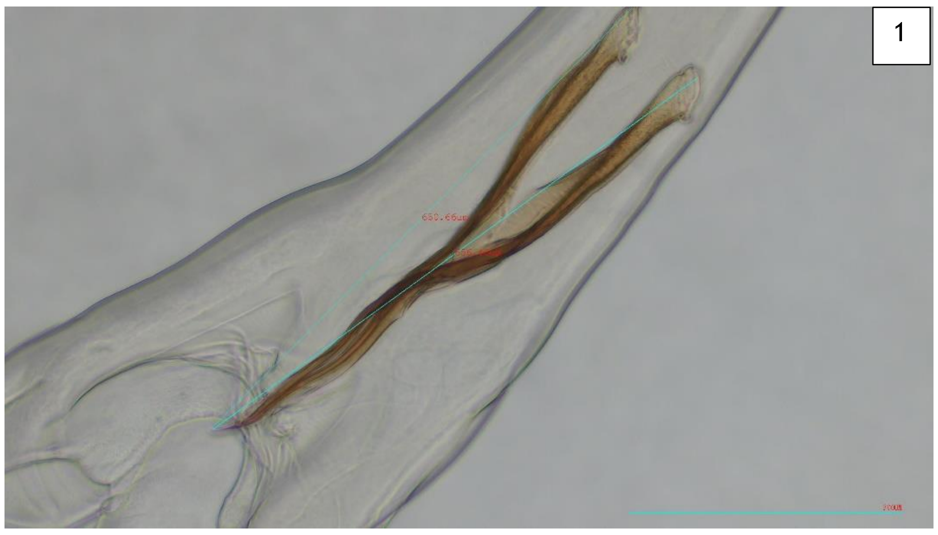


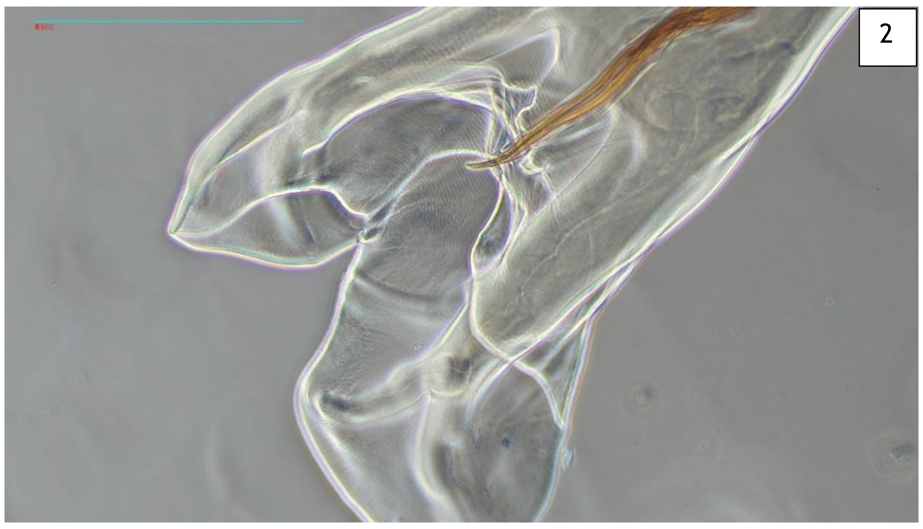


1) Gubernaculum absent. Spicules slender and twisted.
2) Asymmetric bursa with well-developed lateral lobe
