# Supplementary material for: Spiculopteragia boehmi is the dominant abomasal nematode species in reindeer (Nordland County, Norway) sharing pasture with wild and domesticated ruminants
Source: Acta Vet Scand. 2026 Feb 10;68:10. doi: 10.1186/s13028-026-00853-w (PMC12896319; doi:10.1186/s13028-026-00853-w)
Supplement: Supplementary file 3 — Supplementary Material 3 [file 13028_2026_853_MOESM3_ESM.docx]

Additional file. 3. Primers that were used to amplify the targeted Giardia genes by PCR

|  |  | Giardia primers |  |  |  |
| --- | --- | --- | --- | --- | --- |
| Primer name | | Primer sequences 5´- 3´ | Target | Size | Reference |
| First amplification | G7 | AAGCCCGACGACCTCACCCGCAGTG | beta giardin (bg) | 753 bp |  |
|  | G759 | GAGGCCGCCCTGGATCTTCGAGACGAC |  |  |  |
| Second amplification | βGiarF | GAACGAGATCGAGGTCCG |  | 511 bp | [29] |
|  | βGiarR | CTCGACGAGCTTCGTGTT |  |  |  |
| First amplification | GDH1 | TTCCGTRTYCAGTACAACTC | glutamate dehydrogenase (gdh) | 755 bp |  |
|  | GDH2 | ACCTCGTTCTGRGTGGCGCA |  |  |  |
| Second amplification | GDH3 | ATGACYGAGCTYCAGAGGCACGT |  | 530 bp | [30] |
|  | GDH4 | GTGGCGCARGGCATGATGCA |  |  |  |
